# Morphological Evaluation of Meta-stable Oligomers of α-Synuclein with Small-Angle Neutron Scattering

**DOI:** 10.1038/s41598-018-32655-0

**Published:** 2018-09-24

**Authors:** Ghibom Bhak, Soonkoo Lee, Tae-Hwan Kim, Ji-Hye Lee, Jee Eun Yang, Keehyoung Joo, Jooyoung Lee, Kookheon Char, Seung R. Paik

**Affiliations:** 1 Center for Research in Biological Chemistry and Molecular Materials (CIQUS), Organic Chemistry Department, University of Santiago de Compostela (USC), Santiago de Compostela, 15782 Spain; 2 School of Chemical and Biological Engineering, Institute of Chemical Processes, College of Engineering, Seoul National University, Seoul, 08826 Korea; 3 Neutron Science Division, Department of Research Reactor Utilization, Korea Atomic Energy Research Institute, Daejeon, 34057 Korea; 4 Department of Quantum System Engineering, Chonbuk National University, Jeollabuk-do, 54896 Korea; 5 School of Computational Sciences, Korea Institute for Advanced Study, Seoul, 02455 Korea

## Abstract

Amyloidogenesis of α-synuclein (αS) is considered to be a pathological phenomenon related to Parkinson’s disease (PD). As a key component to reveal the fibrillation mechanism and toxicity, we have investigated an oligomeric species of αS capable of exhibiting the unit-assembly process leading to accelerated amyloid fibril formation. These oligomers previously shown to exist in a meta-stable state with mostly disordered structure and unable to seed the fibrillation were converted to either temperature-sensitive self-associative oligomers or NaCl-induced non-fibrillating oligomeric species. Despite their transient and disordered nature, the structural information of meta-stable αS oligomers (Meta-αS-Os) was successfully evaluated with small-angle neutron scattering (SANS) technique. By fitting the neutron scattering data with polydisperse Gaussian Coil (pGC) model, Meta-αS-O was analyzed as a sphere with approximate diameter of 100 Å. Its overall shape altered drastically with subtle changes in temperature between 37 °C and 43 °C, which would be responsible for fibrillar polymorphism. Based on their bifurcating property of Meta-αS-Os leading to either on-pathway or off-pathway species, the oligomers could be suggested as a crucial intermediate responsible for the oligomeric diversification and multiple fibrillation processes. Therefore, Meta-αS-Os could be considered as a principal target to control the amyloidogenesis and its pathogenesis.

## Introduction

α-Synuclein (αS) is an intrinsically disordered protein (IDP) exhibiting a remarkable structural transition to form highly ordered cross β-sheet-based protein aggregates known as amyloid fibrils^1^. Since amyloidogenesis transforms innocuous soluble proteins into toxic insoluble protein aggregates as found in various neurodegenerative disorders including Parkinson’s disease (PD), Alzheimer’s disease (AD), and Prion disease, the protein fibrillation has been intensively studied to unveil its underlying molecular mechanism, which would provide some controlling strategies of the fibrillation for the ultimate development of therapeutics toward the disorders^2^. Nucleation-dependent fibrillation as a widely recognized mechanism requires the preformed nuclei which readily grow into the fibrils through selective accretion of amyloidogenic proteins with a considerable structural adjustment^2,3^. In fact, the oligomeric species of amyloidogenic proteins have been suggested to act as either a nucleation center for the template-dependent fibrillation^4,5^ or a growing unit in the template-independent process as observed with the self-assembly proteins of αS, amyloid-β, and κ-casein^6,7^. Some of the oligomers, however, fail to participate in the fibrillar assembly since they become an off-pathway intermediate^8^. Moreover, since the oligomers have also been suggested to be a pathological culprit responsible for the cellular degeneration, molecular nature of the oligomers and their mutual relationship must be elaborated^6^.

In the case of αS oligomers, however, their structural heterogeneity thwarts detailed investigations. The natively unfolded nature of αS capable of exhibiting structural plasticity upon its self-assembly and multiple partner interactions would be responsible for the multitude of αS oligomers and thus multiple pathways of the fibrillation^9^. We have studied one particular type of meta-stable αS oligomers (Meta-αS-Os) since they act as a growing unit to exhibit the accelerated amyloid fibril formation in the presence of external stimuli such as shear force^10^, temperature change^11^, pH^12^, and organic solvents^13^ which are suspected to alter the structure of Meta-αS-Os into a self-associative state. In fact, this unit-assembly process was confirmed by producing the pea-pod type gold nanoparticle (AuNP) chain aligned within the protein nanofibril of αS from the αS-encapsulated AuNP units (αS-AuNP) in the presence of the stimuli including hexane or pH change^14^. Meta-αS-Os were demonstrated to be a β-sheet free species whose global shape could be readily altered by the external influences for multiple assembly processes leading to the fibrillar polymorphism^11^. In addition, Meta-αS-Os were also shown to be a toxic membrane disrupting agent as they self-assembled into the radiating amyloid fibrils (RAFs) on the surface of liposomes^15^. It was suggested that the *in situ* membrane-dependent fibrillation process, neither the oligomers nor the final amyloid fibrils, might be responsible for the neuronal cell death^15^. It is therefore curious to find out overall appearance of Meta-αS-Os as well as their relationship with the αS oligomers investigated in other studies.

The multitude of αS oligomers could be categorized into either on-pathway or off-pathway intermediate in the fibrillation process^8,9,16–22^. By employing various analytical techniques which include single-molecule fluorescence resonance energy transfer (FRET)^16^, cryo-electron microscopy (cryo-EM)^17^, and small-angle X-ray scattering (SAXS)^8,18,19^, several oligomeric species of αS have been described with their distinctive fibrillation properties. In the single-molecule FRET experiment, two types of αS oligomers were isolated in the early stage of fibrillation process^16^. The first oligomers (type-A) without any defined structure were produced through initial association of monomeric αS molecules, which then converted into the second oligomers (type-B) existing in a compact state with amyloid-like β-sheet conformation. Since the type-B oligomers preceded the amyloid fibrils, the conversion from type-A to on-pathway type-B (type-B_on_) oligomers was considered to be a crucial step for the fibril production. SAXS analysis provided three-dimensional (3-D) structure information of the αS oligomers, which could be possibly classified into type-B_on_ oligomers, as a wreath-like morphology with a dimension of 18 nm × 9 nm × 4.5 nm^19^. However, there has been another species of type-B oligomers which no longer participate in the fibrillation (type-B_off_)^17^. These type-B_off_ oligomers were demonstrated to have a significant level of antiparallel β-sheet conformation which hardly transforms into the parallel β-sheet structure of amyloid fibrils. Cryo-EM analysis indicated that they exist in a doughnut-like 3-D structure with a flat dimension of 14 nm × 10 nm. These oligomers were in a β-sheet rich state^8^ showing considerable stability against the changes in pH and temperature^18^, which would hinder the fibrillation process of monomeric αS. On the basis of SAXS analysis, their shape was suggested to be a prolate ellipsoid with a flat dimension of approximately 14 nm × 9 nm.

To unveil their relationships, we have hypothesized that the unstructured type-A oligomers are a crucial species responsible for the formation of either type-B_on_ or type-B_off_ oligomers as a bifurcation point although a possibility of direct production of type-B_off_ oligomers from αS monomers cannot be completely excluded. From the perspective of structural plasticity based on the molecular characteristics of IDP of αS, the unstructured type-A species could be closely related to our Meta-αS-Os. In this report, the structural information of Meta-αS-Os has been studied with small-angle neutron scattering (SANS) technique, a powerful tool to define protein structures existing especially in a transient and disordered state. Those meta-stable structures usually exhibit a low scattering intensity in SAXS analysis^23^ since proteins mainly comprised of the elements having low electron density such as carbon/hydrogen/oxygen and the transient structures should be examined in a diluted solution condition. The SANS technique, however, is advantageous to obtain higher scattering intensity of those disordered proteins by using deuterium dioxide (D_2_O) that is capable of controlling the scattering contrast between the proteins and solvent. Moreover, low-energy beam of neutron at a level of milli-electronvolt (meV) would cause minimum damage to protein structures. The result shown here is the first example of analyzing the ‘transient’ protein aggregates of αS with SANS technology. This strategy would allow us not only to understand molecular reason(s) for the emergence of oligomeric diversity, but also to suggest an ultimate target for regulation of the pathological fibrillation process.

## Materials and Methods

### Purification of α-synuclein (αS)

αS protein was purified according to the procedure described in the previous paper^2^. In brief, αS was over-expressed in *Escherichia coli* BL21 (DE3), and the heat-treated cell lysate was subjected to DEAE-Sephacel anion-exchange, Sephacryl S-200 size-exclusion, and S-Sepharose cation-exchange chromatography. The purified αS was then stored in aliquots (1 mg/ml) at −80 °C after dialysis against total 6 L of fresh 20 mM Mes at pH 6.5 with two changes over 12 hr.

### Atomic force microscope (AFM) analysis

For the assessment of heat-induced oligomeric unit assembly, an aliquot (10 μl) containing αS oligomers was placed on a cleaved mica surface. Following 30-min incubation at room temperature, the mica was immersed in fresh 20 mM Mes (pH 6.5) and incubated at 80 °C for another 30 min. After the heat-treated mica was cleaned with excessive distillated water, the oligomeric and fibrillar species of αS on the mica were analyzed with AFM (JPK instruments, Germany) in a tapping mode with a Super Sharp tip (SSS-NCHR, NANOSENSORS, Switzerland). To reveal coexistence of the oligomers and fibrils, the αS species (10 μl) was adsorbed on the mica coated with poly-l-lysine for 5 min at room temperature. After the mica was washed with distilled water 5 times and dried in vacuum chamber, AFM analysis was done in a tapping mode.

### Transmission electron microscope (TEM) analysis

Droplet of αS solution (10 μl) was placed onto carbon-coated copper grid (Ted Pella Inc. CA). Following adsorption of αS aggregates, the droplet was displaced with 50 μl of distillated water three times. After staining with 2% uranyl acetate (Electron Microscopy Sciences) for 30 sec, the sample was examined with TEM (JEM 1010, JEOL, Japan).

### Kinetics of αS fibrillation

Fibrillation kinetics of αS was monitored with thioflavin-T (thio-T) binding fluorescence. αS (1 mg/ml) in 20 mM Mes at pH 6.5 was incubated at 37 °C under an agitated condition at 200 rpm. During the incubation, aliquots (20 μl) of αS were mixed with 2.5 μM thio-T (160 μl) in 50 mM glycine at pH 8.5. Then, thio-T binding fluorescence of the samples was measured at an emission wavelength of 482 nm with an excitation at 450 nm by using a luminescence spectrophotometer (LS-55, Perkin-Elmer, CA). The fluorescence intensities plotted versus incubation time were fitted to a sigmoidal curve using Equation (1),

1 $$y={y}_{0}+\frac{a}{1+{e}^{-(\frac{x-{x}_{0}}{b})}}$$

where *y* is the measured fluorescence intensity; *y*_0_ is the initial value of fluorescence; *a* is the maximum intensity; *x*_0_ is the time to reach 50% of *a*; 1/*b* is the apparent first-order rate constant (*k*_app_) for the fibril growth. The time-point for collecting αS oligomers (O_T_) was determined at *x*_0_ – 4*b* when the fluorescence intensity is 1.8% of *a*.

### Circular dichroism (CD) spectroscopy

Protein secondary structure of αS in 20 mM Mes at pH 6.5 was assessed with CD spectroscopy (J-715, Jasco, Japan) scanned between 195 and 250 nm using 0.1-mm path length quartz cell. The CD spectra were obtained as average of five separate scans with step resolution of 1.0 nm, bandwidth of 1.0 nm, and scan speed of 20 nm/min.

### Attenuated total reflectance-Fourier transformation infrared (ATR-FTIR) spectrometer

Differences in the secondary structures of αS present at various states such as monomers, oligomers, and fibrils were evaluated with Nicolet 6700 FTIR spectrometer (Thermo Scientific). The αS samples were located on Zinc Selenide (ZnSe) ATR crystal, and then the ATR-FTIR spectra were monitored with the spectrometer equipped with DTGS detector at a spectral resolution of 4 cm^−1^.

### Small-angle neutron scattering (SANS) measurements

SANS experiments were performed using the 40-m SANS instrument at HANARO (High-flux Advanced Neutron Application Reactor) in KAERI (Korea Atomic Energy Research Institute). SANS measurements were done with neutrons at 6 Å of wavelength with a full width at half maximum of 12%, and the sample-to-detector distances (SDD) of 5.5 m and 1.16 m were employed to monitor the *q* range of 0.009 Å^−1^ < *q* < 0.67 Å^−1^. In the SANS experiments, all the samples were dissolved in D_2_O to enhance the neutron scattering contrast between the proteins and solvent.

### Analysis of SANS data

SANS intensities were presented as *I*(*q*), the differential scattering cross section per unit volume, where q is the scattering wave vector described by Equation (2),

2 $$q=\frac{4{\rm{\pi }}\,\sin (\theta /2)}{\lambda }$$

Here *λ* is the wavelength of neutrons and *θ* is a magnitude of the scattering angle. The sample scattering was corrected with background, empty cell scattering, and sensitivity of individual detector pixels. The datasets were corrected in an absolute scale by using a software for the data reduction provided by HANARO through the direct beam flux method. And SANS intensities were analyzed with IGOR pro software^24^.

Scattering length density (SLD) of αS is given by Equation (3),

3 $${\rm{SLD}}\,{\rm{of}}\,{\rm{\alpha }}S={b}_{{\rm{m}}}/{V}_{{\rm{m}}}$$

where *b*_m_ is the sum of scattering lengths of all the amino acids in αS proteins (140 residues: MDVFMKGLSK_10_AKEGVVAAAE_20_KTKQGVAEAA_30_GKTKEGVLYV_40_GSKTKEGVVH_50_GVATVAEKTK_60_EQVTNVGGAV_70_VTGVTAVAQK_80_TVEGAGSIAA_90_ATGFVKKDQL_100_GKNEEGAPQE_110_GILEDMPVDP_120_DNEAYEMPSE_130_EGYQDYEPEA_140_) and *V*_m_ is the sum of dry volume of each amino acid. Both parameters of *b*_m_ and *V*_m_ were obtained from a report providing the values of scattering lengths of amino acid residues^23^. Assuming that all hydrogen atoms were exchanged with D_2_O solvent, the SLD of αS was calculated as 3.02 × 10^−6^ Å^−2^. For the detailed structural analysis of the protein, the non-linear least squares model fits were conducted by using polydisperse Gaussian Coil (pGC)^25^ and Triaxial Ellipsoid (TE)^26^ model functions. The pGC model, which is based on the calculation of an empirical functional form for scattering from a polydisperse polymer chain in a solvent. The polymer has a Schulz-Zimm polydispersity, then the returned value is scaled to unit of [cm^−1^ sr^−1^] on absolute scale. The scattering intensity *I*(*q*) is calculated by Equation (4),

4 $$I(q)=scale\frac{2[{(1+Ux)}^{-1/U}+x-1]}{(1+U){x}^{2}}+{\rm{bkg}}$$

where the dimensionless chain dimension is given by Equation (5),

5 $$x=\frac{{R}_{g}^{2}{q}^{2}}{1+2U}$$

and the polydispersity is given by Equation (6),

6 $$U=\frac{{M}_{w}}{{M}_{n}}-1$$

where *U* = Polydispersity, bkg = background, *Rg* = radius of gyration, *Mw* = weight average molecular weight, Mn = number average molecular weight.

Assuming the overall structure of polymer is a sphere, its volume (*V*) is given by Equation (7),

7 $$V=\frac{4\pi }{3}{(\sqrt{\frac{5}{3}}{R}_{g})}^{3}$$

The TE model calculates the form factor for a triaxial ellipsoid with uniform scattering length density (SLD). The form factor is normalized by the particle volume given by Equation (8),

8 $${\rm{P}}({\rm{q}})=scale\,\frac{ < {f}^{2} > }{Vol}+bkg$$

where < > is an average over all possible orientations of the ellipsoid. An instrument resolution smeared version is also provided. The function calculated is for an ellipsoid where all three semi-axes are of different lengths. For the results of the calculation to be valid, the axes must be defined as: a ≤ b ≤ c

then *P*(*q*) is calculated by Equation (9),

9 $$P(q)=\frac{scale}{{V}_{el}}{\int }_{0}^{1}{\int }_{0}^{1}{\varnothing }^{2}\{q{[{a}^{2}co{s}^{2}(\frac{\pi x}{2})+bsi{n}^{2}(\frac{\pi x}{2})(1-{y}^{2})+{c}^{2}{y}^{2}]}^{\frac{1}{2}}\}dxdy$$

where the function $\varnothing (x)$ is defined by Equation (10)

10 $${\varnothing }^{2}(x)=9{(\frac{sinx-xcosx}{{x}^{3}})}^{2}$$

The volume of the ellipsoid is given by Equation (11)

11 $${V}_{el}=\frac{4\pi }{3}abc$$

And its radius of gyration given by Equation (12)

12 $${R}_{g}^{2}=\frac{{a}^{2}+{b}^{2}+{c}^{2}}{5}$$

The returned value is in units of [cm^−1^] on absolute scale.

### Preparation of monomeric αS in deuterium dioxide (D_2_O)

Lyophilized αS monomers were obtained by using freeze dryer (FDU-2000, Sunil Eyela, Japan) after dialyzing the purified αS in 20 mM Mes at pH 6.5 against total 6 L of distilled water. Completely freeze-dried αS was dissolved in 5 mM Mes (pH 6.5) made with D_2_O solvent (Cambridge Isotope Laboratories, MA). The buffer pH was adjusted with sodium deuteroxide (NaOD) (Cambridge Isotope Laboratories, MA).

### Filtration-induced facilitated fibrillation of αS oligomers

The repetitive centrifugal membrane filtrations of αS oligomers were conducted by using Omega Nanosep-30 (PALL) with Centrifuge 5415 R (Eppendorf, Germany) at 14,000 *g* at 37 °C. Following each filtration for 2 min, the filtrate was combined to the original sample and subjected to another round of the filtration. The resulting oligomers and amyloid fibrils were collected by resuspending them with the same initial volume of 20 mM Mes (pH 6.5). NaCl solution was added to each sample in 20 mM Mes (pH 6.5) to adjust the final concentrations of αS (1 mg/ml) and NaCl (150, 15, 1.5, 0.15, and 0.015 mM).

## Results and Discussion

### Formation of Meta-αS-Os

**Figure 1:**
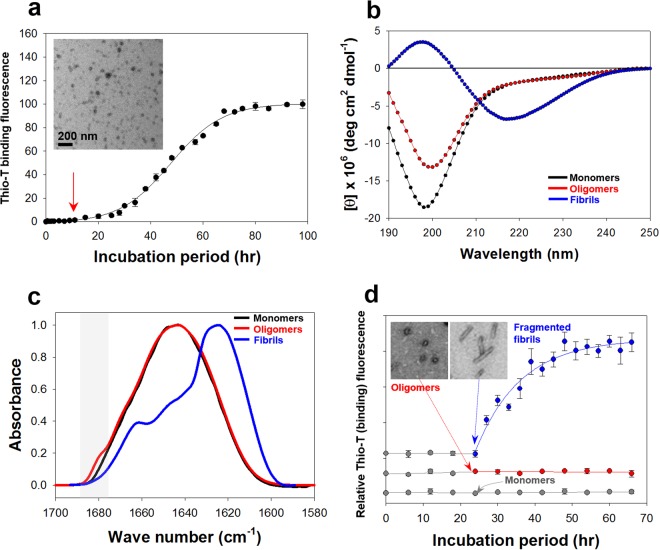
Formation of Meta-αS-Os. (**a**) Kinetics of αS fibrillation monitored with thio-T binding fluorescence under an agitated incubation of αS. Time-point of the Meta-αS-Os formation is indicated by a red arrow. TEM image of Meta-αS-Os is provided in inset. (**b**) CD and (**c**) ATR-FTIR spectra of monomers (black), oligomers (red), and fibrils (blue) of αS. (**d**) Fibrillation kinetics of monomeric αS seeded with either monomers (gray), oligomers (red), or fragmented fibrils (blue) at a fixed concentration of 5% under a quiescent incubation. Time-points of the seed addition are indicated by arrows with the corresponding colors. TEM images of oligomers and short fibrils are presented in inset.

Meta-αS-Os exhibiting the unit-assembly leading to accelerated amyloid fibril formation were collected from the early lag phase of the αS fibrillation process monitored with thioflavin-T (thio-T) binding fluorescence, a specific assay for detecting β-sheet structure of amyloid fibrils (Fig. 1a). The lack of thio-T binding fluorescence distinguishes the oligomers from mature fibrils as a discrete species in the process of protein self-assembly. Transmission electron microscope (TEM) revealed that the oligomers existed as homogeneous spheres with an average diameter of 18 nm (Fig. 1a, inset). Based on our previous result, this oligomer comprises approximately 11 monomers as analyzed with the static light scattering (SLS) data^10^. To obtain the oligomers satisfying the strict conditions of spherical homogeneity in the absence of any β-sheet content, the oligomer collection time (O_T_) was determined as the time-point reaching 1.8% of the maximal fluorescence intensity in the fibrillation kinetics (see Materials and Methods)^11^. For αS at three different concentrations of 1, 3, and 5 mg/ml, the oligomers were consistently obtained at O_T_ points of 9, 3, and 0.5 hr, respectively (Fig. S1). These oligomers were, however, not stable enough to survive size-exclusion chromatography (data not shown)^10^ since they were found to be readily dissociated into monomers under the non-equilibrating condition, indicating that the oligomers exist in a meta-stable state. Circular dichroism (CD) analysis suggested that Meta-αS-Os exist mainly in a disordered state by showing a typical spectrum of random structure protein with a single minimum ellipticity at 197 nm. The minimum level, however, increased from that of monomeric αS, a well-known member of intrinsically disordered proteins (IDPs) (Fig. 1b), indicating that the oligomers may have certain local structures. In fact, a previous study employing the single-molecule fluorescence experiment suggested that αS oligomers were not in a state of simple random structure as determined with FRET analysis^16^. Additional assessment with the attenuated total reflectance-Fourier transform infrared (ATR-FTIR) also confirmed that the secondary structure of Meta-αS-Os was almost identical to that of αS monomers, showing the same major peak at 1650 cm^−1^ indicative of the disordered/random structure (Fig. 1c) whereas the amyloid fibrils with parallel β-sheet conformation usually yield the major peak at 1620 cm^−1^. Interestingly, the FTIR spectrum of the oligomers also showed a small distinct shoulder peak at 1680 cm^−1^, indicating the presence of anti-parallel β-sheet structures, which could be contributed possibly by a trace amount of type-B_off_ oligomers^17^. Owing to high energy barrier for the conversion from anti-parallel to parallel β-sheet structure, the anti-parallel β-sheet-containing type-B_off_ oligomers were demonstrated to be inefficient for the fibrillar extension. Consistently, our oligomers were not able to nucleate the αS fibrillation in the presence of monomeric αS whereas the fragmented fibrils facilitated the process (Fig. 1d). Taken together, Meta-αS-Os have been shown to exist in a spherical form under a meta-stable state with mostly random structure, which are not capable of seeding the amyloid fibril formation.

### Transformation of Meta-αS-Os into either on-pathway or off-pathway intermediate

**Figure 2:**
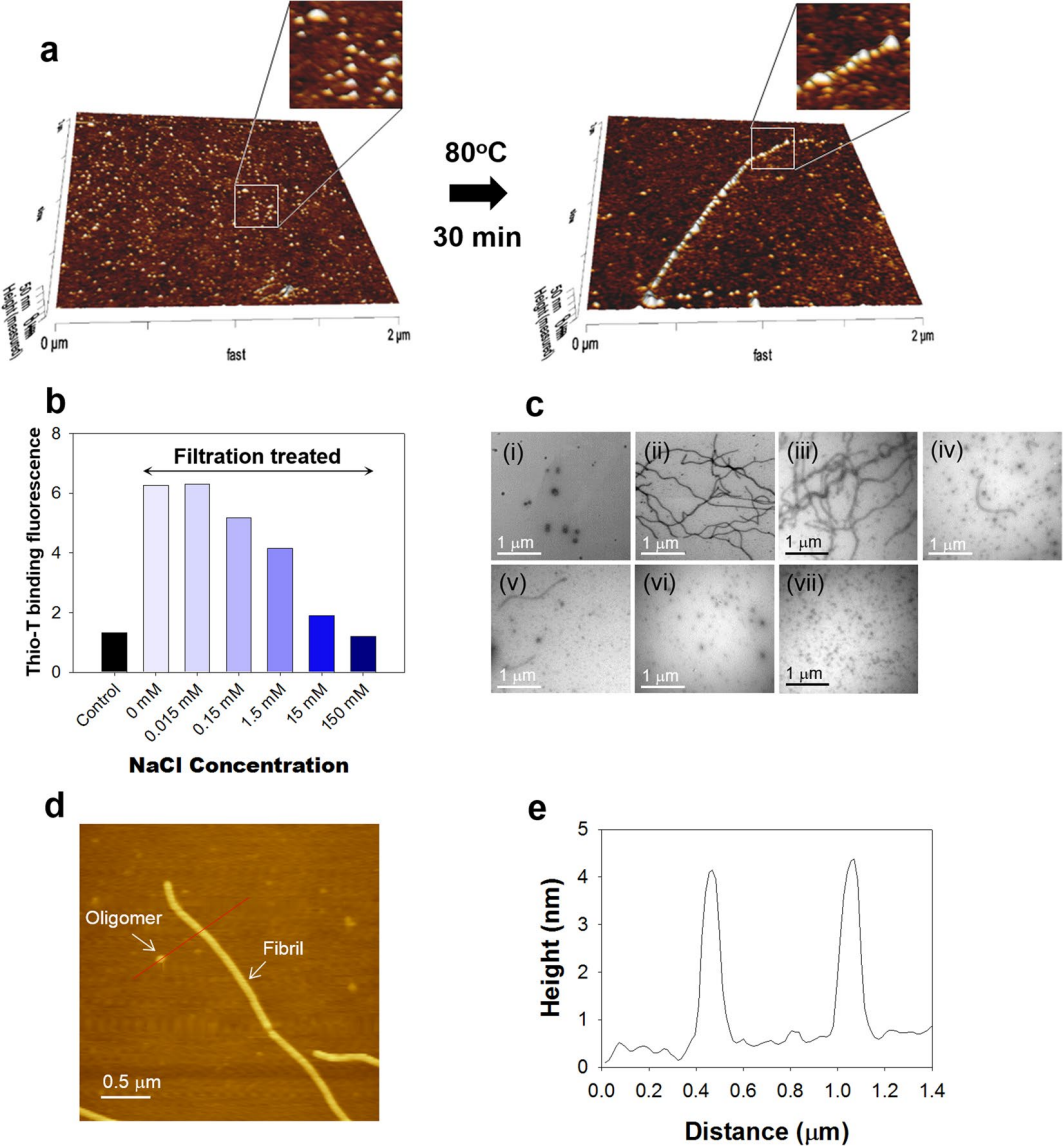
Unit-assembly of Meta-αS-Os. (**a**) 3-D AFM images of the scattered Meta-αS-Os species and an extended fibrillar structure on the mica surface. Black arrow represents the heat treatment at 80 °C for 30 min. (**b**) Thio-T binding fluorescence of αS after the centrifugal membrane filtration in the absence or presence of NaCl at various concentrations of 0, 0.015, 0.15, 1.5, 15, and 150 mM. (**c**) TEM images of the αS oligomers before (i) and after the membrane filtration at various concentrations of 0 (ii), 0.015 (iii), 0.15 (iv), 1.5 (v), 15 (vi) or 150 mM (vii). (**d**) AFM image showing coexistence of the oligomers and the amyloid fibrils at 1.5 mM NaCl following the membrane filtration. (**e**) AFM height profile of the oligomeric and fibrillar species.

Instead, Meta-αS-Os have played a growing unit for the unit-assembly-based facilitated amyloid fibril formation. Their unit-assembly was examined on the surface of mica immersed in 20 mM Mes (pH 6.5) at 80 °C for 30 min. In the 3-D AFM images, the scattered Meta-αS-Os on the mica surface became aligned with each other to form the extended fibrils following the heat treatment (Fig. 2a). This phenomenon reflects general two-step process of protein-surface interaction showing initial reversible binding of the oligomers to the surface followed by their irreversible stabilization into the amyloid fibrils via the transition of Meta-αS-Os into type-B_on_ oligomers upon the heat treatment. In addition, the Meta-αS-Os were also shown to be converted into type-B_off_ oligomers in the presence of NaCl. Without NaCl, the centrifugal membrane filtration of Meta-αS-Os gave rise to a dramatic increase in thio-T binding fluorescence, indicating instant transformation of the oligomers into amyloid fibrils (Fig. 2b) as demonstrated in the previous reports^10,27^. The resulting amyloid fibrils were revealed with TEM (Fig. 2c-ii) whereas the oligomers remained unaltered without the membrane filtration (Fig. 2c-i). In the presence of NaCl, however, the enhanced thio-T binding fluorescence of the oligomers observed with the membrane filtration decreased considerably as the salt concentration increased to 150 mM (Fig. 2b). The TEM images also indicated that the fibrils were found to be less populated while another group of granular species became abundant as the NaCl concentration increased (Fig. 2c-iii to c-vii). The result clearly suggested that the oligomer-to-fibril conversion was obviously suppressed by NaCl with an emergence of the particulate species reflecting type-B_off_ oligomers less prone to convert into the amyloid fibrils. AFM analysis revealed that the particulates and the short fibrils of αS obtained with 1.5 mM NaCl had almost identical height profile with the maximum height of 4 nm (Fig. 2d,e). Taken together, Meta-αS-Os have been shown to be able to convert into two different types of the oligomeric species - self-associative type-B_on_ oligomers and non-fibrillating type-B_off_ oligomers.

### Shape analysis of Meta-αS-Os with SANS

**Figure 3:**
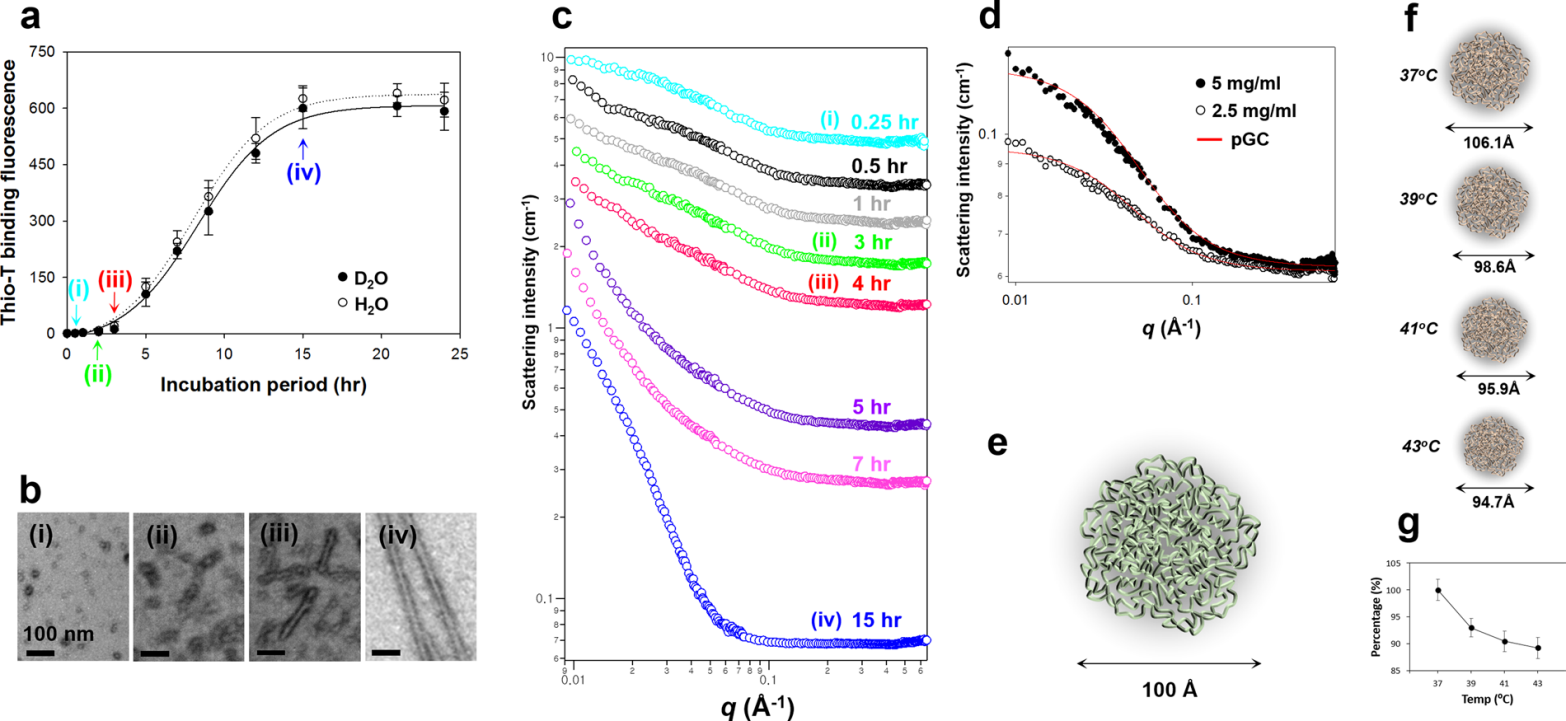
SANS analysis of Meta-αS-Os. (**a**) Kinetics of amyloid fibril formation of αS (5 mg/ml) in 5 mM Mes (at pH 6.5) containing either D_2_O (closed circles) or H_2_O (open circles). Aliquots of the aggregates were collected at four different time points of (i) 0.25 hr, (ii) 3 hr, (iii) 4 hr, and (iv) 15 hr. (**b**) TEM images of (i) oligomers, (ii) associated oligomers, (iii) short fibrils, and (iv) mature fibrils. (**c**) SANS intensities of the αS aggregates obtained at different incubation time points of 0.25 hr (cyan), 0.5 hr (black), 1 hr (gray), 3 hr (green), 4 hr (red), 5 hr (violet), 7 hr (pink), and 15 hr (blue). SANS intensities are vertically shifted to clearly show the differences in their SANS patterns. (**d**) SANS spectra of Meta-αS-Os at the protein concentration of either 2.5 (open dots) or 5.0 (closed dots) mg/ml. The red solid lines are the theoretical fits to the polydisperse Gaussian Coil (pGC) model. (**e**) Structure of Meta-αS-O schematized based on the SANS analysis. The diameter of 100 Å is indicated. (**f**) Schematic representation of the Meta-αS-O structures obtained at different temperatures of 37 °C, 39 °C, 41 °C, and 43 °C, with diameters of 106.1 Å, 98.6 Å, 95.9 Å, and 94.7 Å, respectively. (**g**) Plot of temperature-dependent decline percentage of the diameters.

To investigate global shape of Meta-αS-Os in solution with SANS, the αS aggregates were prepared in deuterium dioxide (D_2_O) to enhance their scattering contrast. The fibrillation kinetics of αS (5 mg/ml) dissolved in 5 mM Mes∙D_2_O showed a typical sigmoid curve identical to that observed in 5 mM Mes∙H_2_O (Fig. 3a). The protein aggregates prepared in D_2_O were collected at four different time points of (i) 0.25 hr at O_T_ in the beginning of lag phase, (ii) 3 hr in the middle of lag phase, (iii) 4 hr in the end of lag phase, and (iv) 15 hr in the final stationary phase (Fig. 3a), which were revealed with TEM as (i) isolated spheres of Meta-αS-Os, (ii) associated spheres, (iii) short fibrils, and (iv) mature fibrils (Fig. 3b), respectively. In the early stage of lag time, the SANS intensity is low and the low-*q* part is flat, which is a typical pattern of random coils or spherical nanostructures^28,29^ (Fig. 3c,i). Then, low-*q* region turned into *q*^−1^ behavior indicative of cylindrical structure as the incubation time increased^28^ (Fig. 3c, ii–iv), suggesting that unstructured oligomeric spheres converted into amyloid fibrillar structures in the aggregation process. For the Meta-αS-Os species, their neutron scattering *I*(*q*) at two different concentrations of 2.5 mg/ml and 5 mg/ml were acquired in a *q* range from 0.009 to 0.67 Å^−1^ (Fig. 3d).

To obtain the detailed structural information, the non-linear least squares model fits using numerical functions were carried out. Solid red lines were the best theoretical fits to a polydisperse Gaussian Coil (pGC) model, showing *Chi*^2^ values of 497.7 and 493.0 at two different concentrations of 2.5 and 5 mg/ml, respectively. Meta-αS-Os were analyzed as a spherical form with diameter of 100.4 (±3.1) Å (Fig. 3e) with a $\sqrt{{x}^{2}/N}$ value of 1.5 and 100.6 (±2.6) Å with a $\sqrt{{x}^{2}/N}$ value of 1.5 at the concentrations of 2.5 and 5 mg/ml, respectively (Table S1). The volume of Meta-αS-Os was obtained as approximately 530 (±49.1) nm^3^ (Table S1) by calculating with the equation of sphere volume (see Materials and Methods). Meta-αS-Os were estimated to be about 18-mer when the volume of oligomers was divided by that of αS molecule^19^. In fact, we had previously reported that the oligomers were calculated as 11-mer from the analysis with SLS^10^. Since the SLS was used to obtain the molecular weight of αS oligomers via Debye plot analysis, the number of monomers comprising an oligomer was estimated by simply dividing the molecular weight of oligomer (159 kDa) with a calculated molecular weight of αS monomer (14.4 kDa). Considering compacting effect of monomers upon the oligomer formation, this evaluation might be underestimated as the experimental data was evaluated with the theoretical value. Therefore, the difference in the number of monomers between those two studies would result from the different size evaluation of the oligomers in terms of either the molecular weight (SLS) or the particle volume (SANS).

To confirm accuracy of the pGC model fitting, a comparative analysis was also conducted with another fit of Triaxial Ellipsoid (TE) model (Fig. S2). While the pGC model is based on an empirical functional form of a polydisperse polymer chain, the TE model is given as a form factor for an average over all possible orientations of an ellipsoid. In comparison with the pGC model, the TE model-based data fitting actually provided more detailed information of Meta-αS-O structure, showing that three different radii (R_1_, R_2_, and R_3_) of the ellipsoid obtained at 5 mg/ml were 84.1 (±1.5) Å, 24.4 (±0.7) Å, and 5.7 (±0.9) Å, respectively. However, the rod-like shape determined with the TE model was not consistent with the spherical morphology examined with TEM. In addition, the ellipsoid (rigid structure)-based TE model yielded a lower population of Meta-αS-Os to approximately 25% while the pGC model showed the population of about 68% as a spherical amorphous soft structure with the remainder for non-scattering soluble species. Relationships between fitted volume fraction and αS oligomer average particle volume were used to determine the fraction of oligomers. Considering non-scattering species in solution as monomers, since αS monomer did not show any scattering pattern by SANS. In pGC model, scale factor value, average particle volume, scattering length density (SLD) of αS and deuterium dioxide (D_2_O) were used to calculate volume fraction using Equation (13),

13 $$\frac{d{\rm{\Sigma }}}{d{\rm{\Omega }}}(0)={\rm{\phi }}{V}_{p}{{\rm{\Delta }}{\rm{\rho }}}^{2}$$

where φ is the volume fraction of particles (φ = N_p_V_p_), V_p_ is the average particle volume, and Δρ^2^ is the scattering length density contrast squared^30^. On the other hand, in TE model, scale value, which is equal to volume fraction of sample, was divided by αS concentration to calculate proportion of oligomers. Given that Meta-αS-Os are a structurally disordered species, therefore, the fitting analysis with the pGC model should provide a reliable information to portray the meta-stable oligomeric structure. The results obtained with the SANS experiments are summarized in Table S1. Since the data sets collected at two different concentrations (2.5 and 5 mg/ml) are almost identical, the Meta-αS-Os structure has been suggested to be independent of the protein concentration.

**Figure 4:**
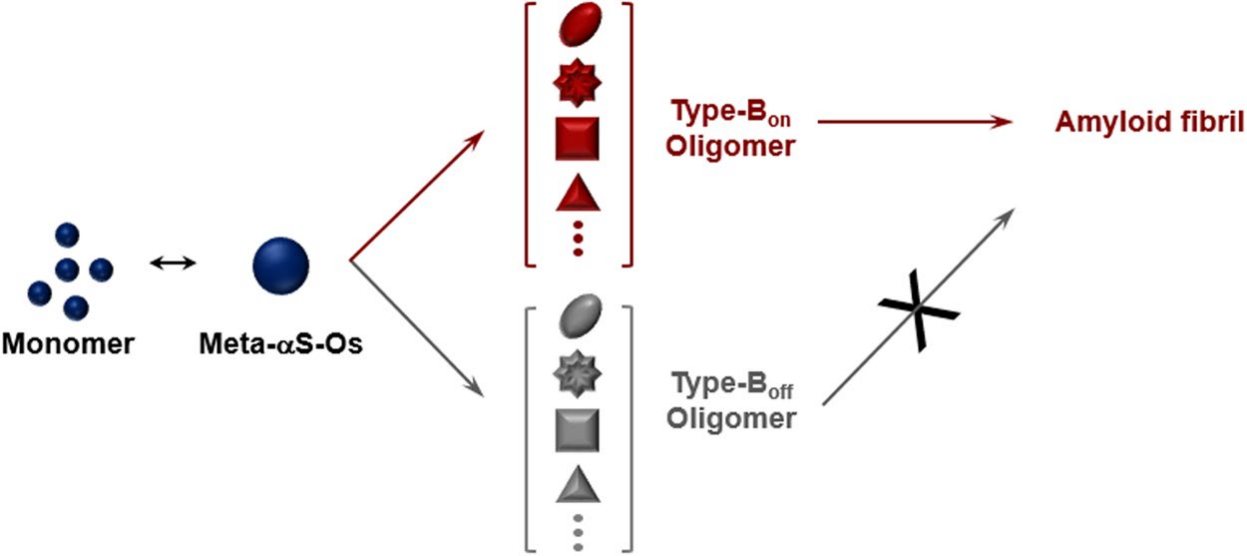
Inter-relationship of the multiple species of αS oligomers classified into either type-B_on_ or type-B_off_ from a bifurcating species of Meta-αS-Os.

In addition, Meta-αS-Os are demonstrated to be susceptible to environmental stimuli by altering its overall shape since the oligomers exhibited considerable structural flexibility in a disordered state. With *in situ* SANS measurement of the oligomers in a thermostat adjusted to several fixed temperatures, Meta-αS-Os altered their structure with subtle changes in temperature ranging from 37 °C to 43 °C at 2 °C intervals. Based on the pGC model, the diameter of Meta-OαS decreased successively as the incubation temperature increased: 106.1 (±2.1) Å at 37 °C, 98.6 (±1.8) Å at 39 °C, 95.9 (±2.1) Å at 41 °C and 94.7 (±2.1) Å at 43 °C (Figs 3f, S3 and Table S2) with *Chi*^2^ values of 621.7, 605.1, 640.4 and 846.2, respectively. Decline percentages of the diameter were 7% from (37 °C to 39 °C), 9.6% (37 °C to 41 °C), and 10.8% (37 °C to 43 °C) (Fig. 3g). In the TE model, as the temperature was elevated, the value of R_1_ decreased: 85.8 (±1.1) Å at 37 °C, 79.3 (±1.1) Å at 39 °C, 77.3 (±1.1) Å at 41 °C, and 76.6 (±1.1) Å at 43 °C (Fig. S4 and Table S3). Decline percentages of the R_1_ value were 7.6% (37 °C to 39 °C), 9.8% (37 °C to 41 °C), and 10.7% (37 °C to 43 °C). This temperature-dependent shape change of Meta-αS-Os was consistent with our previous study demonstrating that the oligomer structure was altered at molecular level by responding to the physiological/pathological temperatures ranging from 37 °C to 43 °C as monitored with the tyrosine intrinsic fluorescence and the 8-anilino-1-naphthalenesulfonic acid (ANS) binding fluorescence^11^. It is, therefore, pertinent to consider that the structural alteration of Meta-αS-Os examined with SANS has resulted from the molecular rearrangement within the oligomer. This conformational change of Meta-αS-Os that would produce a self-associative form of type-B_on_ oligomers, which is responsible for the oligomeric unit assembly leading to amyloid fibril formation as observed in various conditions such as hexane treatment^13^, membrane filtration^10,27^, and mild heat-treatment^11^. In the presence of NaCl, however, the Meta-αS-Os turned into type-B_off_ oligomers. Therefore, the structural plasticity of Meta-αS-Os present mainly in a disordered state is responsible for its conformational rearrangement responding to external stimuli, which would bifurcate or further diversify their assembly property in the fibrillation process (Fig. 4). Taken together, Meta-αS-Os have been suggested to be a crucial early species capable of being converted into various forms of oligomers in terms of their structure, size, and stability depending on particular physiological/pathological conditions. Although it is not clear whether Meta-αS-Os are identical to type-A oligomers, those oligomers derived from Meta-αS-Os can be categorized into multiple species exhibiting either type-B_on_ or type-B_off_ oligomers with differential cytotoxicity, suggesting a possible existence of multiple parallel pathways of αS oligomers for their contributions in the fibrillation processes and toxic consequences. Meta-αS-Os, therefore, could be considered as an important target not only to understand the mechanism of amyloidogenesis and the toxic oligomer formation but also to design therapeutic strategies toward PD.

## Electronic supplementary material

Supporting Information
