# Supplementary material for: Morphological Evaluation of Meta-stable Oligomers of α-Synuclein with Small-Angle Neutron Scattering
Source: Sci Rep. 2018 Sep 24;8:14295. doi: 10.1038/s41598-018-32655-0 (PMC6155208; doi:10.1038/s41598-018-32655-0)
Supplement: Supplementary file 1 — Supporting Information [file 41598_2018_32655_MOESM1_ESM.docx]

**Supporting Information**

**Morphological Evaluation of Meta-stable Oligomers of α-Synuclein with Small-Angle Neutron Scattering**

Ghibom Bhak^1,†^, Soonkoo Lee^2,†^, Tae-Hwan Kim^3,4^, Ji-Hye Lee^2^, Jee Eun Yang^2^,

Keehyoung Joo^5^, Jooyoung Lee^5^, Kookheon Char^2^, and Seung R. Paik^2^*

^1^Center for Research in Biological Chemistry and Molecular Materials (CIQUS), Organic Chemistry Department, University of Santiago de Compostela (USC), Santiago de Compostela 15782, Spain

^2^School of Chemical and Biological Engineering,

College of Engineering, Seoul National University, Seoul 08826, Korea

^3^Neutron Science Division, Department of Research Reactor Utilization,

Korea Atomic Energy Research Institute, Daejeon 34057, Korea

^4^Department of Quantum System Engineering, Chonbuk National University,

Jeollabuk-do, Korea

^5^School of Computational Sciences, Korea Institute for Advanced Study,

Seoul 02455, Korea

^†^These authors equally contributed to this work.

**Supporting Figures**


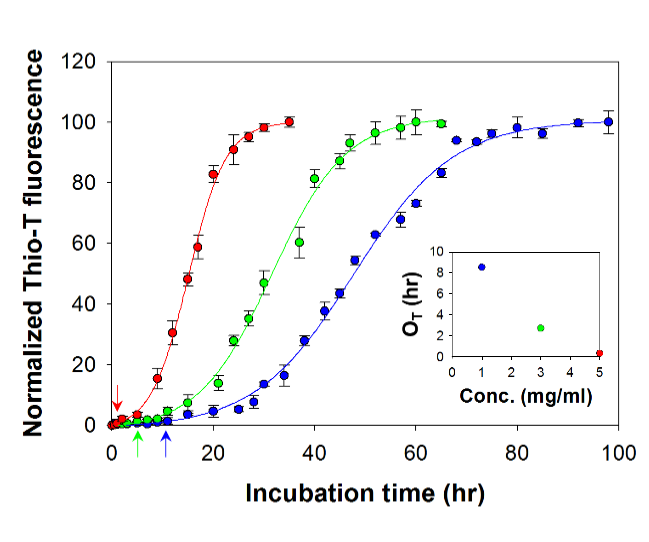


**Figure S1. Time-point for Meta-αS-O collection (O_T_).** The O_T_ at three different concentrations of 1 (blue), 3 (green), and 5 (red) mg/ml are indicated by arrows. A plot of O_T_ versus αS concentrations is provided in inset.

**
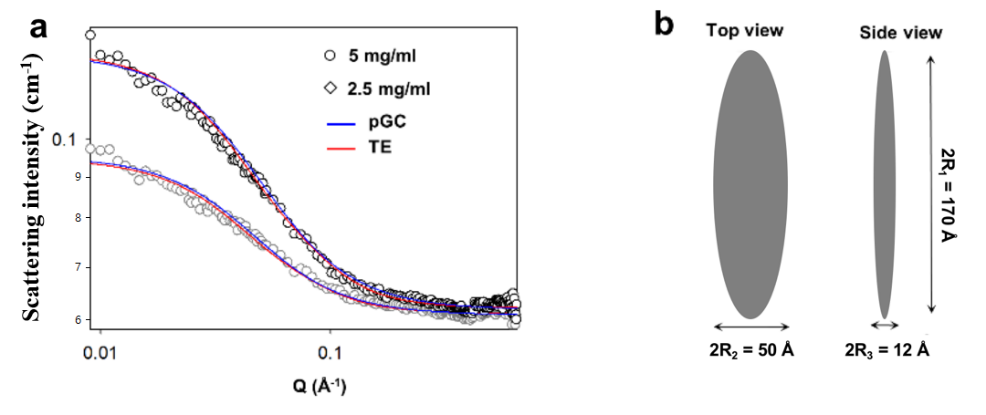
**

**Figure S2. Analysis of SANS data with triaxial ellipsoid (TE) model.** (a) SANS intensities of Meta-αS-Os at the protein concentration of either 2.5 (diamond) or 5.0 (circle) mg/ml. Red solid lines are the theoretical fits to TE model. For the comparison, the fits to polydisperse Gaussian coil (pGC) model are in blue. (b) Schematic representation of Meta-αS-O obtained via the analysis with TE model. (See Table S1 for details)


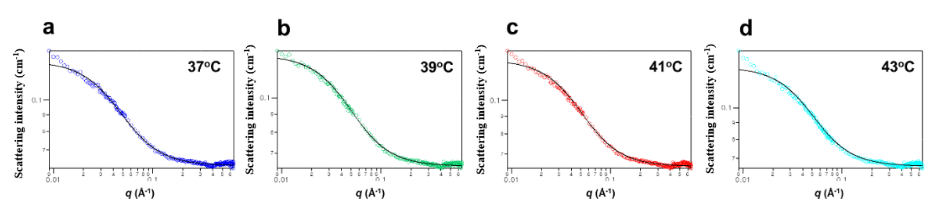


**Figure S3. SANS intensities of Meta-αS-Os at four different temperatures of (a) 37^o^C, (b) 39^o^C, (c) 41^o^C, and (d) 43^o^C.** Black solid lines are the theoretical fits to pGC model.

**
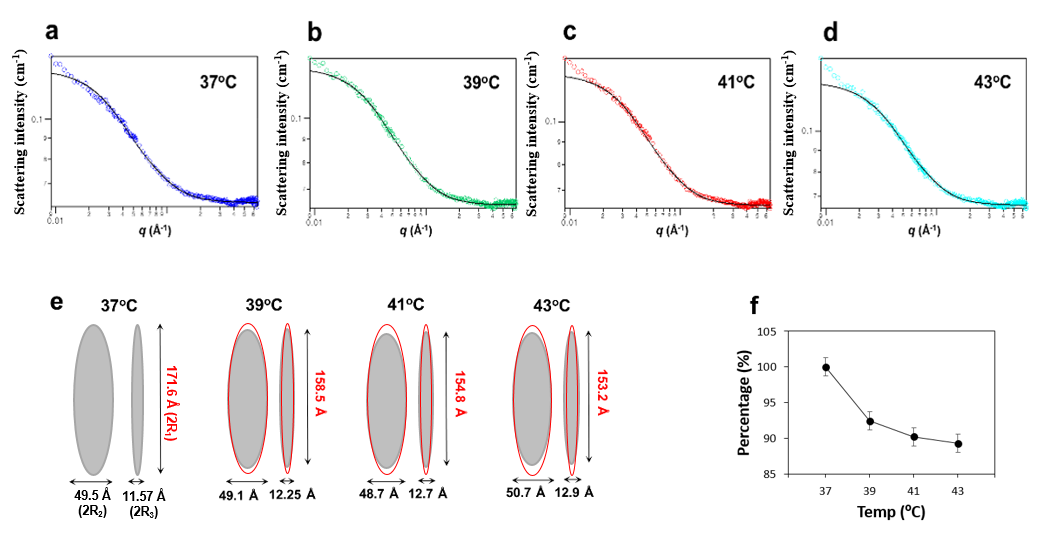
**

**Figure S4. Structural changes monitored by SANS analysis with TE model.** SANS spectrum of Meta-αS-Os measured at (a) 37^o^C, (b) 39^o^C, (c) 41^o^C, or (d) 43^o^C. (e) Diagrams of temperature-dependent changes in the Meta-αS-O structure. (f) Plot for decline percentages of R_1_. (See Table S3 for details)

**Table S1. Results of two model fitting analyses for SANS data of Meta-αS-O**

**
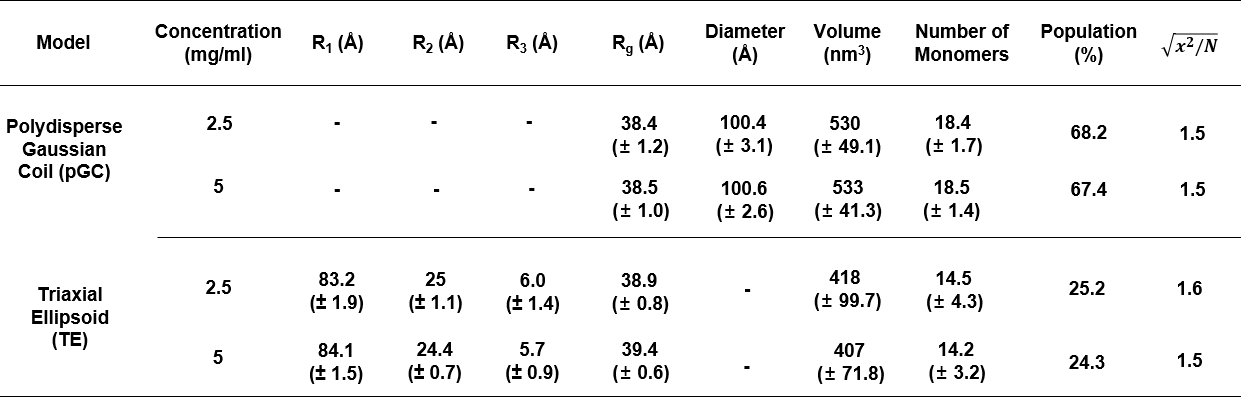
**

**Table S2. Parameters of pGC model fitting to SANS data of Meta-αS-O depending on temperature**

**
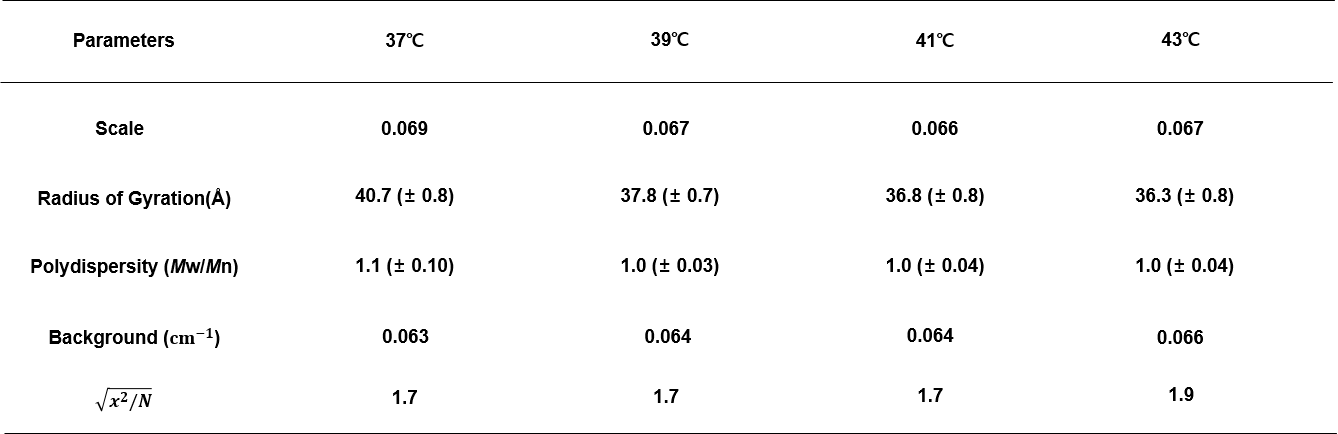
**

**Table S3. Parameters of TE model fitting to SANS data of Meta-αS-O depending on temperature**

**
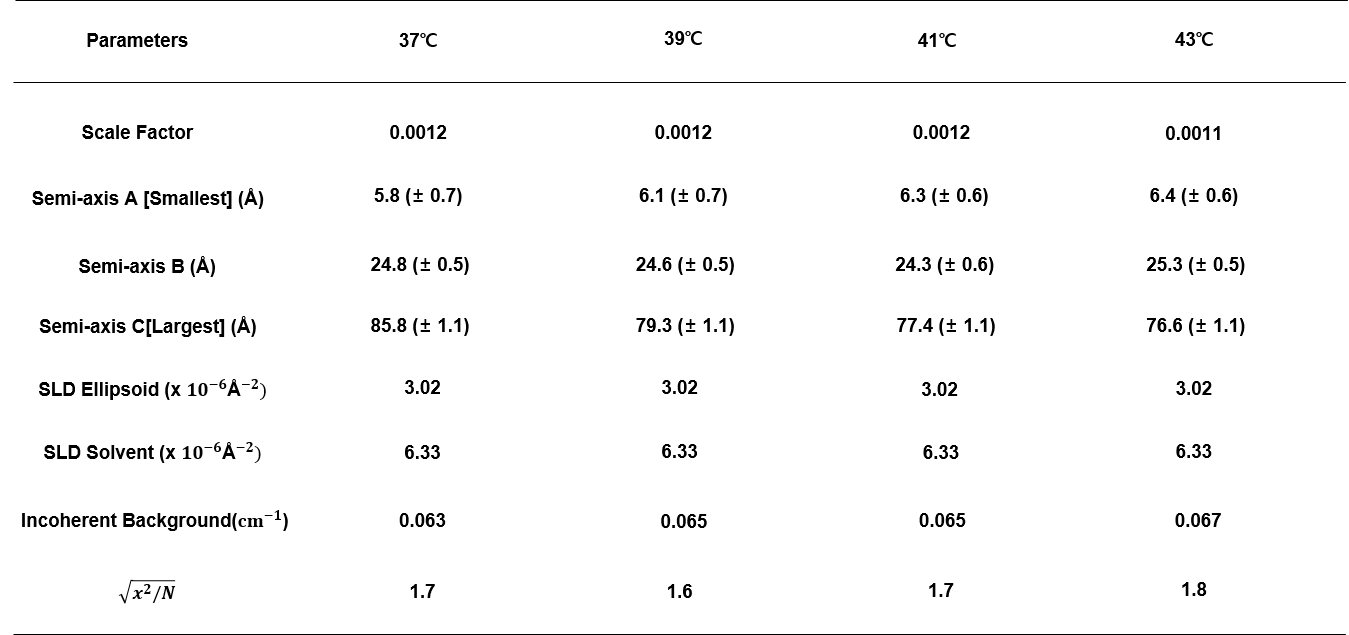
**
